# Local environment in biopsy better predict the pathological response to neoadjuvant chemoradiotherapy in rectal cancer

**DOI:** 10.1042/BSR20190003

**Published:** 2019-03-26

**Authors:** Yan Huang, Xiao-ying Lou, Ya-xi Zhu, Yu-chen Wang, Lei Zhang, Hai-ling Liu, Chao Wang, Huan-miao Zhan, Zhi-qiang Cheng, Wei-yan Tan, Lei Wang, Xin-juan Fan

**Affiliations:** 1 Department of Pathology, The Sixth Affiliated Hospital of Sun Yat-sen University, Guangzhou 510655, China; 2 Department of Colorectal Surgery, The Sixth Affiliated Hospital of Sun Yat-sen University, Guangzhou 510655, China; 3 Department of Orthopedics, Guangzhou Huaxin Orthopedic Hospital, Guangzhou, China; 4 Department of Biliary-Pancreatic Surgery, Guangdong Provincial Key Laboratory of Malignant Tumor Epigenetics and Gene Regulation, Sun Yat-sen Memorial Hospital, Sun Yat-sen University, Guangzhou, China

**Keywords:** rectal cancer, neoadjuvant chemoradiotherapy, tumor-infiltrating lymphocytes, tumor budding, tumor regression grade

## Abstract

Neoadjuvant chemoradiotherapy (nCRT) followed by surgery is the standard treatment for locally advanced rectal cancer. Here, we analyzed the impact of local and systemic environments on the tumor response to preoperative chemoradiotherapy in rectal cancer. We recruited 141 patients with rectal cancer treated with nCRT. We evaluated the local tumor environment, including tumor-infiltrating lymphocytes (TILs), intratumor budding (ITB), and the systemic inflammatory environment, including the neutrophil-to-lymphocyte ratio (NLR) and C-reactive protein (CRP) level. Our finding revealed that tumor regression was significantly associated with the density of CD8+ TILs in the intraepithelial, the presence of ITB, the combination of NLR and CRP (NLR-CRP) value, and the combination of CD8+ intraepithelial TIL (iTIL) density and ITB presence. Moreover, multivariate analysis showed that only the combination of CD8+ iTILs and ITB was an independent predictive factor for the pathological response to nCRT in rectal cancer. Our finding demonstrate that the local tumor environment was a better predictor of the tumor response than the systemic environment and thus provided new insight into screening for patients who are more likely to benefit from cancer treatment.

## Introduction

Colorectal cancer remains one of the leading causes of cancer death worldwide [1]. In patients with locally advanced rectal cancer (stage II and III), neoadjuvant chemoradiotherapy (nCRT) could significantly reduce the local recurrence and treatment-associated toxicity, and more importantly, make tumors more amenable to resection [2]. However, the tumor response to nCRT varies greatly among patients. Only 8–14% of patients attain a pathologic complete response (PCR), and watchful waiting is proposed [3]. Approximately 20% of these patients will not respond to nCRT and will even suffer significant side effects and miss their best opportunity for surgery. Evidence of the correlation between PCR and both long-term disease-free survival and overall survival is emerging [4]. Therefore, finding effective approaches for predicting the treatment response and further selecting populations who are likely to benefit most from nCRT is urgent.

More recently, the complex role of the tumor microenvironment has been widely studied and shown to impede the tumor response to chemoradiotherapy [5]. In particular, tumor-infiltrating lymphocyte (TIL) markers, such as CD4, CD8, CD45RO, and FOXP3, and intratumor budding (ITB) have been identified as markers predicting the pathological response to nCRT in rectal cancer [6–11]. However, the reported predictive values of each marker vary between previous studies. For example, Yasuda et al. [6] reported that a high density of CD8+ T lymphocytes in tumor biopsy samples was strongly correlated with the tumor reduction ratio and was an independent factor for achieving a complete response after nCRT; conversely, McCoy et al. [12] found that CD8+ T cells had no significant association with tumor regression grade (TRG). Similarly, the reported predictive values of tumor budding vary [13,14]. This discrepancy might arise in part because these studies focused only on one or several aspects of the tumor environment, but cancer progression may also be controlled by the body’s systemic inflammatory responses.

The prognostic value of the systemic inflammatory response has been studied in many tumors [15]. High leukocyte numbers, neutrophil-to-lymphocyte ratios (NLRs) and C-reactive protein (CRP) levels have been associated with poorer outcomes in many cancers, such as testicular germ cell tumors, squamous cell carcinoma, soft tissue sarcoma, and colorectal cancer [16–19]. A dysregulated systemic inflammatory response could promote cancer progression and further influence the therapeutic effect of chemo- or chemoradiotherapy.

In the present study, we assessed the local and systemic tumor environments and further examined their predictive value relative to nCRT response. For the local environment, we evaluated the density of intraepithelial TILs (iTILs), stromal TILs (sTILs), and ITB in biopsy samples. Meanwhile, the systemic inflammatory environment, including the NLR and CRP levels, was also examined. Furthermore, we analyzed the correlations between the local and systemic environments. Our study revealed that the local tumor environment was a better predictor of the tumor response to nCRT than was the systemic environment and thus provided new insight into understanding the pathological response to neoadjuvant therapy.

## Materials and methods

### Study cohort

As shown in Supplementary Figure S1, 141 pathologically confirmed rectal cancer patients who accepted nCRT and underwent subsequent rectal resection were recruited from January 2012 to December 2017 in the Department of Pathology of the Sixth Affiliated Hospital of Sun Yat-sen University. The tumor was clinically assessed for the T and N stages by pelvic magnetic resonance imaging (MRI) and for the M stage by computed tomography (CT) of the thorax, abdomen, and pelvis. The clinical/serological variables, such as: age; gender; tumor stage; levels of carcinoembryonic antigen (CEA), carbohydrate antigen 19-9 (CA19-9), and CRP; and the NLR, were obtained prior to treatment.

Patients with distant metastases at the time of diagnosis or with Lynch syndrome, ulcerative colitis or familial adenomatous polyposis were excluded. All patients underwent a tumor biopsy before nCRT and subsequent rectal resection. The patients were treated according to the recommendations of the National Comprehensive Cancer Network (NCCN) guidelines (version 3, 2017) and underwent pelvic radiotherapy (dose range, 45–55 Gy) with concurrent 5-fluorouracil-based chemotherapy (FOLFIRI or FOLFOX regimens). Our study was approved by the Clinical Ethics Review Committee of the Sixth Affiliated Hospital of Sun Yat-sen University. Clinical informed consent was obtained from all patients.

### Serological variables

Routine blood tests were performed within 7 days before nCRT inception. Hematological parameters, including lymphocyte and monocyte counts as well as CEA, CA19-9, and CRP levels, were measured. The NLR was calculated as the neutrophil count divided by the lymphocyte count. All serological parameters were divided into two subgroups by level (high vs low). In addition, the combination of NLR and CRP (NLR-CRP) was used to classify patients into the NLR-CRP high (high levels of NLR and/or CRP) and NLR-CRP low (low levels of both NLR and CRP) groups.

### Immunohistochemical staining

Formalin-fixed, paraffin-embedded rectal biopsy tissue specimens were cut into 4 μm sections for immunohistochemical staining. The immunohistochemical staining was performed according to the following protocol. First, sections were deparaffinized three times in xylene for 20 min and rehydrated with a graded alcohol series (100% ethyl alcohol for 3 min, 95% ethyl alcohol for 3 min, and 75% ethyl alcohol for 3 min). Slides were immersed in antigen retrieval solution (sodium citrate, pH 6.0), heated in a microwave for 15 min and incubated in H_2_O_2_ for 10 min. Slides were then washed in a PBS buffer for 10 min and blocked with normal goat serum at 37°C for 20 min. Sections were incubated overnight at 4°C in humidified chambers with primary antibodies against CD4 (1:250; ZSGB-BIO, Beijing, China) and CD8 (1:500; ZSGB-BIO, Beijing, China) and were then washed in PBS buffer three times for 5 min. Next, sections were treated with a secondary antibody for 30 min at room temperature and incubated with a 3,3’-diaminobenzidine (DAB) chromogen for 10 min. Finally, sections were washed in PBS buffer for 10 min and lightly counterstained with hematoxylin to reveal nuclei. A negative control was generated by replacing the specific primary antibody with nonimmune serum immunoglobulins.

### TIL assessment

**Figure 1:**
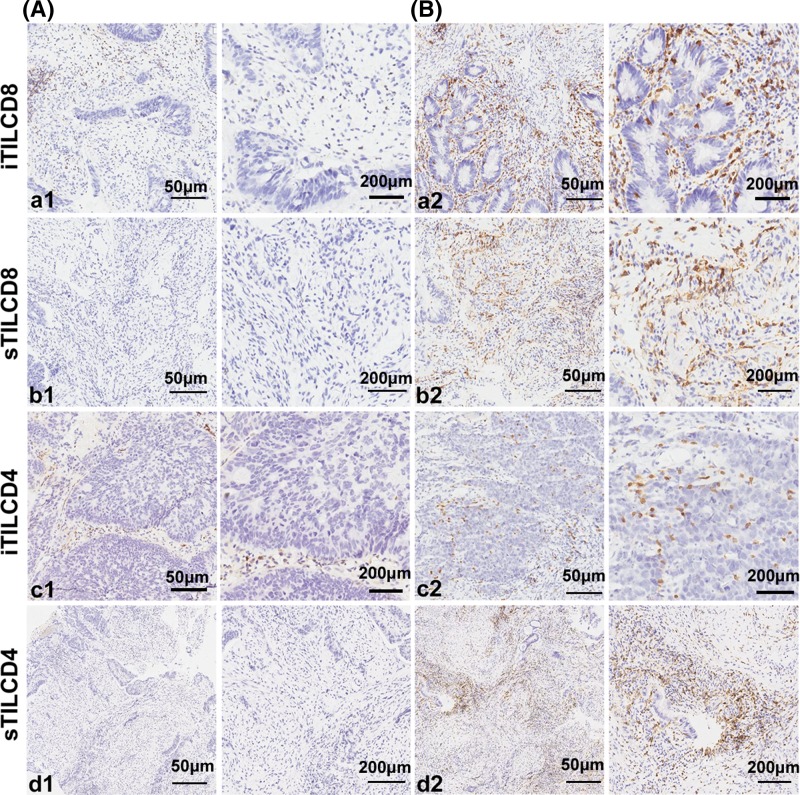
The expression of CD8 and CD4 in rectal cancer Immunohistochemical staining of CD8+ and CD4+ T cells in biopsy samples of rectal cancer before nCRT at a low density (**A**; left panels, ×100; right panels, ×400) and a high density (**B**; left panels, ×100; right panels, ×400). In each subgroup, the right panels display a representative enlarged view from the area shown in the left panels. The iTILCD8 (a1, a2) and sTILCD8 (b1, b2). The iTILCD4 (c1, c2) and sTILCD4 (d1, d2). One representative staining example is shown. Abbreviations: iTILCD4, tumor-infiltrating CD4+ lymphocytes in the intraepithelial; iTILCD8, tumor-infiltrating CD8+ lymphocytes in the intraepithelial; sTILCD4, tumor-infiltrating CD4+ lymphocytes in the stromal compartment; sTILCD8, tumor-infiltrating CD8+ lymphocytes in the stromal compartment.

We evaluated the density of both iTILs and sTILs by immunohistochemical staining, as shown in Figure 1. Both CD4 and CD8 were clearly stained in the cell membrane. The number of immunoreactive lymphocytes in different locations was counted separately. For iTILs, we counted the number of immunoreactive lymphocytes in cancer cell nests at ×400 magnification. Three fields with the greatest abundance of TILs were selected to ensure representativeness and homogeneity. Similarly, for sTILs, we selected the three fields with the greatest abundance of immunoreactive lymphocytes in the tumor stroma and counted the number of immunoreactive lymphocytes at ×400 magnification. If the amount of biopsy tissue was insufficient for three fields, one or two fields were evaluated. The densities of TILs were independently assessed by two gastrointestinal pathologists who were blinded to all clinical and pathological data (X.Y.L. and Y.X.Z.). For the adjacent tissue, lymphocytes showed CD4+ or CD8+. The distribution of positive cells depends on the densities of lymphocytes.

### Tumor budding

**Figure 2:**
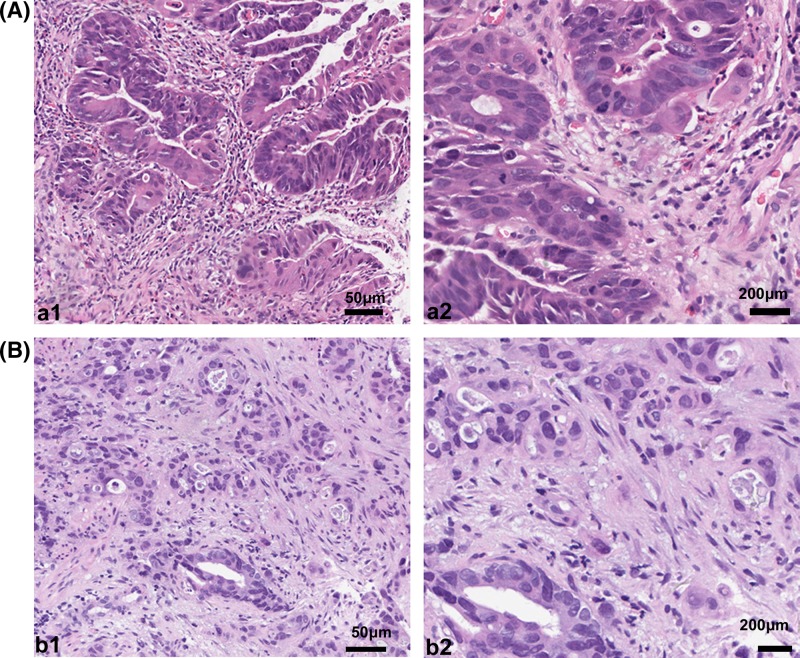
H&E-stained biopsy samples of rectal cancer before nCRT Rectal cancer without tumor budding (a1, ×100; a2, ×400) and with tumor budding (b1, ×100; b2, ×400)

Tumor budding was assessed on hematoxylin and eosin (H&E)-stained slides archived at the Institute of Pathology, the Sixth Affiliated Hospital of Sun Yat-sen University. All of the H&E sections were stained with automatic H&E dyeing machine (Thermo, ygz-cl-D052). Tumor budding in biopsy tissue was defined as the presence of a single cancer cell or of small clusters of fewer than five cells in the tumor stroma. In the biopsy sections, tumor budding was detected at ×100 magnification and confirmed at ×400 magnification. An example of tumor budding is shown in Figure 2. The presence of tumor budding was evaluated independently by two pathologists (X.Y.L. and Y.X.Z.).

### Tumor regression grade (TRG)

The TRG of a primary tumor after nCRT was evaluated on H&E-stained slides based on the 7th edition of the American Joint Committee on Cancer and the Union for International Cancer Control (AJCC/UICC) systems. TRGs were defined as follows: grade 0, complete response, no remaining viable cancer cells; grade 1, moderate response, only small clusters or single cancer cells remaining; grade 2, minimal response, residual cancer remaining with predominant fibrosis; and grade 3, poor response, minimal or no tumor killing with extensive residual cancer. Patients with TRGs of 0 and 1 were considered the good response cohort; those with TRGs of 3 and 4, the poor response cohort. All histological slides were independently reviewed by two gastrointestinal pathologists (X.Y.L. and Y.X.Z.).

### Statistical analysis

Statistical analyses were performed using SPSS software v. 17.0 (SPSS, Inc., Chicago, IL, U.S.A.). Receiver operating characteristic (ROC) curve analysis was used to determine the cutoff values of the hematological parameters and TIL densities. In this analysis, we established the cutoff value with the maximum sensitivity and specificity for predicting the TRG. The cutoff values for the hematological parameters and TIL densities are shown in Table 2. Associations between the clinical/immunological variables and the TRG were assessed by a chi-square test. Multivariate analyses were performed using backward stepwise variable selection. Differences and associations were considered statistically significant for *P*<0.05.

## Results

### Clinicopathological characteristics

A total of 141 patients with rectal cancer were studied. The detailed characteristics of the patients are listed in Table 1. The median age was 54 years (range 21−86 years), and the vast majority (70%) of the patients were male. In addition, 21% (30/141) of the patients were clinically evaluated as stage II; 79% (111/141), stage III. The frequency of TRGs 0, 1, 2, and 3 were *n*=27, *n*=34, *n*=61, and *n*=19, respectively. Overall, 43% (61/141) of the patients had a good response to nCRT.

**Table 1:** Clinicopathological characteristics of patients

	*n*=141
Age, mean (SD)	54
Sex, *n* (%)	
Male	99 (70%)
Female	42 (30%)
TNM stage, (*n*%)	
Stage II	30 (21%)
Stage III	111 (79%)
TRG, (n%)	
0	27 (19%)
1	34 (24%)
2	61 (43%)
3	19 (14%)
Presence of ITB	
Yes	26 (18%)
No	115 (82%)

### TIL density in biopsy tissues and its associations with TRG

The number and distribution of TILs in biopsy samples obtained before nCRT were evaluated. CD4+ and CD8+ TILs were distributed mainly in the stroma (average 77 [5–300] and 42 [6–120], respectively) compared with their distribution in intraepithelial areas (average 0.46 [0–12] and 7.31 [0–53], respectively) (Figure 1). CD8+ iTILs were detected in 89% (126/141) of the cases, while only 11% (15/141) of the cases demonstrated positive staining for CD4+ iTILs.

We next examined the associations between the density of the TILs and the pathological response. As shown in Table 2, CD8+ iTILs were significantly correlated with tumor regression (*P*<0.01). The densities of CD8+ iTILs were substantially higher in the good response cohort. In addition, the regression of tumors with a high density of CD8+ sTILs tended to be better (51%) than that of tumors with a low CD8+ sTIL density (36%), although statistical significance was not reached (*P*=0.080). No significant differences in the density of either CD4+ iTILs or CD4+ sTILs were observed between the good response and poor response cohorts (*P*=0.054 and *P*=0.140, respectively).

**Table 2:** Correlation of TRG with TILs and serological markers

	Response to nCRT		*P*-value
	Good (*n*=61)	Poor (*n*=80)	
Age			
≥60	22	31	0.744
<60	39	49	
Gender			
Male	45	54	0.420
Female	16	26	
TNM stage			
Stage II	9	21	0.098
Stage III	52	59	
Presence of ITB			
Yes	7	19	0.048*
No	54	61	
iTILCD4			
≥1	3	12	0.54
<1	58	68	
sTILCD4			
≥41	37	40	0.208
<41	27	40	
iTILCD8			
≥4.5	48	38	<0.001*
<4.5	13	42	
sTILCD8			
≥ 42.5	35	34	0.080
<42.5	26	46	
CEA			
≥2.84	22	44	0.026*
<2.8	39	36	
CA199			
≥7.15	37	61	0.046*
<7.15	24	19	
NLR			
≥2.48	8	18	0.083
<2.48	53	62	
CRP			
≥2.74	19	33	0.145
<2.74	42	47	
NLR-CRP			
High	21	43	0.022*
Low	40	37	
iTILCD8+ and ITB 2 groups			
iTILCD8 high and without ITB	40	21	<0.001*
iTILCD8 high and with ITB/	24	56	
iTILCD8 low and without ITB/			
iTILCD8 low and with ITB			

* *P*<0.05. Abbreviations: CA199, carbohydrate antigen 199; iTILCD4, tumor-infiltrating CD4+ lymphocytes in the intraepithelial; iTILCD8, tumor-infiltrating CD8+ lymphocytes in the intraepithelial; sTILCD4, tumor-infiltrating CD4+ lymphocytes in the stromal compartment; sTILCD8, tumor-infiltrating CD8+ lymphocytes in the stromal compartment.

### Tumor budding in biopsy tissues and its association with TRG

Tumor budding was identified in 26 of the 141 cases (18%). As shown in Table 2, the pathological response was significantly associated with tumor budding; patients with tumor budding in their biopsy tissues tended to have a poorer response to nCRT than did patients without tumor budding (27 vs 47%, *P*=0.048).

### Serological factors and their associations with TRG

As shown in Table 2, significant correlations were found between the levels of both CEA and CA19-9 and tumor regression (*P*=0.026 and *P*=0.046, respectively). The levels of CEA and CA19-9 were lower in the good response cohort than in the poor response cohort. However, neither the NLR nor the CRP level was significantly different between the two groups (*P*=0.083 and *P*=0.145, respectively). Furthermore, we combined the NLR and the CRP into a single variable and found a significant difference in tumor regression between the NLR-CRP-high group and the NLR-CRP-low group (*P*=0.022).

### Correlations between the local and systemic environments

In the NLR-CRP-low group, the CD8+ sTIL and CD4+ sTIL densities were significantly associated with tumor regression (*P*=0.043 and *P*=0.032, respectively) (Table 3). Furthermore, we analyzed the density of CD8+ sTILs and CD4+ sTILs in the NLR-CRP-high group, but no significant correlations with tumor regression were found (*P*=0.659 and *P*=0.790, respectively) (Table 4).

**Table 3:** The sTILCD8 and sTILCD4 in NLR-CRP low group (*n*=77)

	Response to nCRT		*P*-value
	Good (*n*=40)	Poor (*n*=37)	
sTILCD8			
High	24	14	
Low	16	23	0.043*
sTILCD4			
High	28	1	0.32*
Low	12	20	

Abbreviations: sTILCD4: tumor infiltrating CD4+ lymphocytes in stromal compartment; sTILCD8: tumor infiltrating CD8+ lymphocytes in stromal compartment.

* *P*<0.05.

**Table 4:** The sTILCD8 and sTILCD4 in NLR-CRP high group (*n*=64)

	Response to nCRT		*P*-value
	Good (*n*=40)	Poor (*n*=37)	
sTILCD8			
High	11	20	0.659
Low	10	23	
sTILCD4			
High	11	21	0.790
Low	10	22	

Abbreviations: sTILCD4: tumor infiltrating CD4+ lymphocytes in stromal compartment; sTILCD8: tumor infiltrating CD8+ lymphocytes in stromal compartment.

### Local environment in biopsy tissues and its association with TRG

The combination of TIL densities and ITB in biopsies could represent the local tumor environment. In the subgroup with a higher density of CD8+ iTILs and no ITB (*n*=61), 66% (40/61) of the patients had a good response to nCRT. Univariate analysis revealed a significant correlation between the local environment and the TRG (*P*<0.001) (Table 2).

### Multivariate analysis

To study the potential independent relationship among variables with respect to the TRG, a multivariate regression analysis was performed. As shown in Table 5, only the combination of CD8+ iTILs and ITB was an independent factor related to nCRT response (HR: 0.273; 95% CI: 0.095–0.451; *P*=0.003).

**Table 5:** Multivariate analyses for predictors associated with TRG

	OR	95% CI	*P*-value
Age	−0.006	−0.173 to 0.160	0.941
Sex	0.075	−0.099 to 0.249	0.395
TNM stage	0.099	0.300 to 0.101	0.329
sTILCD4	0.051	−0.115 to 0.216	0.546
CA199	−0.096	−0.278 to 0.086	0.298
CEA	−0.106	−0.274 to 0.016	0.211
NLR	−0.001	−0.288 to 0.286	0.993
CRP	−0.006	−0.387 to 0.374	0.974
NLR-CRP	−0.136	−0.567 to 0.295	0.534
sTILCD8	0.052	−0.128 to 0.231	0.570
iTILCD8+ and ITB	0.273	0.095 to 0.451	0.003*

* *P*<0.05.Abbreviations: CA199, carbohydrate antigen 199; sTILCD4: tumor infiltrating CD4+ lymphocytes in stromal compartment; sTILCD8: tumor infiltrating CD8+ lymphocytes in stromal compartment.

## Discussion

The nCRT has been proved very beneficial for patients with rectal cancer. In the present study, we evaluated the predictive value of both the local tumor microenvironment and the systemic inflammatory response, and we further examined the correlations between the local and systemic environments. Tumor microenvironment-related variables, including the CD8+ iTIL density and ITB, and systemic inflammatory response-related factors (i.e., NLR-CRP), were significantly correlated with tumor regression. Furthermore, multivariate regression analysis showed that only the combination of CD8+ iTIL density and ITB was an independent factor for tumor regression, thus indicating that the local tumor environment is a better predictor of the pathological response to nCRT than is the systemic inflammatory response.

Rectal tumors are composed of two cellular compartments: the malignant epithelium and the stroma. Recently, TILs and ITB have been associated with the tumor response to preoperative chemoradiotherapy [13,20]. However, the previous studies reported different results [12]. In our study, each of these factors affected tumor regression differently. The CD8+ iTIL density and ITB were significantly correlated with the tumor response to nCRT (*P*<0.001 and *P*=0.048, respectively). However, no correlations between CD8+ sTILs or CD4+ sTILs and tumor regression (*P*=0.080 and *P*=0.208, respectively) were observed. Some studies have reported that the density of CD4+ TILs was significantly associated with the nCRT response [6]. The results were thought to differ because the effect of local CD4+ TILs may not be an independent predictive factor and may be influenced by other factors.

We further analyzed the associations between the treatment response and the presence of an elevated systemic inflammatory response. Unlike previous studies, our study indicated that neither CRP nor NLR was associated with tumor regression [21,22]. When we combined these two inflammatory factors into a new variable, namely, NLR-CRP, we found a significant association between NLR-CRP and tumor regression (*P*=0.022). To our knowledge, our research is the first to propose a combination of different inflammatory factors (NLR and CRP) for predicting the response to nCRT in rectal cancer.

Furthermore, we investigated the correlations between the local and systemic environments. Interestingly, we found that CD4+ sTILs and CD8+ sTILs demonstrated significant predictive value in the NLR-CRP-low group, while in the NLR-CRP-high group, these factors were not significantly associated with the TRG; these results suggest that the effects of CD4+ sTILs and CD8+ sTILs could be influenced by the systemic inflammatory response. Our results showed the importance of tumor–host interactions and further determine the oncological outcomes.

Importantly, our multivariate analysis revealed a combined biomarker (CD8+ sTIL density and ITB) that was indeed a stronger predictor of the tumor response than were other variables, thus suggesting that the local environment contributed more heavily to the tumor response than did the systemic environment. The reason for this effect might be that chemo- and radiotherapy-mediated necrotic tumor cells could induce the release of tumor antigens and facilitate the recruitment of immune cells, and the activated CD8+ T cells could then kill tumor cells directly [23,24]. The local antitumor immunity was enhanced after nCRT by the increased numbers of CD8+ TILs. Meanwhile, ITB was reported to be linked to epithelial–mesenchymal transition (EMT) [25,26], which is a critical mechanism for cancer progression and is correlated with chemo- or chemoradiotherapy response [27,28].

The present study had several limitations. First, the study cohort was recruited from one center; thus, validation in a larger multicenter cohort is warranted. Second, the heterogeneity of TILs in the tumor remains a complicated issue, and we evaluated three different fields to reduce the effect of this issue. Furthermore, we investigated ITB in biopsy tissues rather than at the tumor’s invasive front. The value of tumor budding as a predictive marker in biopsies is limited by its diagnostic reproducibility. Finally, the associations between the above characteristics and long-term outcomes, such as disease-free survival and overall survival, were not analyzed. However, these relationships are being examined in further ongoing studies.

In conclusion, we assessed the characteristics of the local and systemic environments, and we further analyzed the effect of these characteristics on the tumor regression response to nCRT. Our results indicated that a high density of CD8+ TILs and the absence of tumor budding were significantly correlated with a good response. Furthermore, multivariate analysis showed that this combined biomarker was indeed a predictive factor, thus indicating that the local environment is a better predictor of the pathological response to nCRT than is the systemic environment.

## Supporting information

**Supplementary Figure 1:** 

## Abbreviations

CA19-9: carbohydrate antigen 19-9
CEA: carcinoembryonic antigen
CRP: C-reactive protein
H&E: hematoxylin and eosin
ITB: intratumor budding
iTIL: intraepithelial TIL
nCRT: neoadjuvant chemoradiotherapy
NLR: neutrophil-to-lymphocyte ratio
NLR-CRP: combination of NLR and CRP
PCR: pathologic complete response
TIL: tumor-infiltrating lymphocyte
TNM: tumor-node-metastasis
TRG: tumor regression grade
sTIL: stromal TIL
