# Supplementary material for: Local environment in biopsy better predict the pathological response to neoadjuvant chemoradiotherapy in rectal cancer
Source: Biosci Rep. 2019 Mar 26;39(3):BSR20190003. doi: 10.1042/BSR20190003 (PMC6434387; doi:10.1042/BSR20190003)
Supplement: Supplementary file 1 [file bsr-39-bsr20190003_Supp1.pdf]

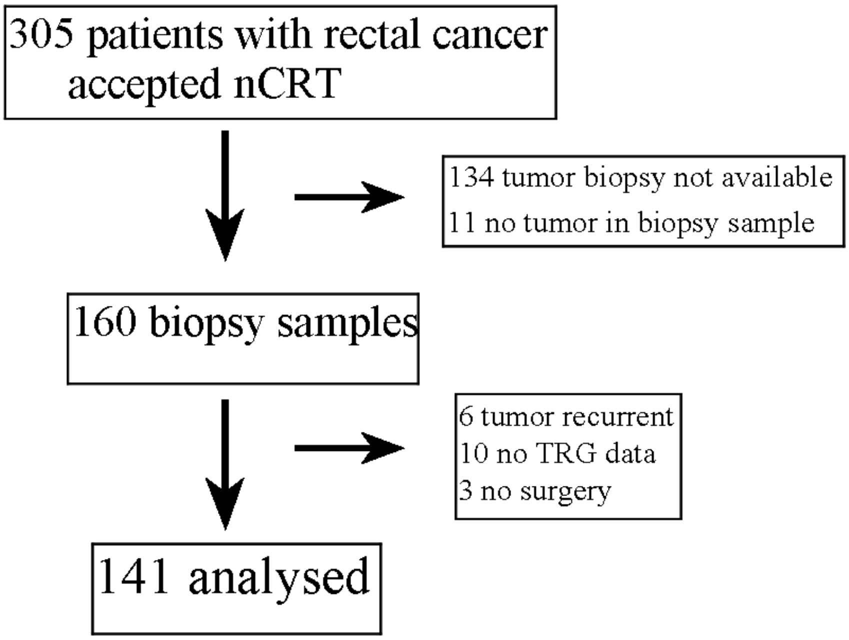
305 patients with rectal cancer accepted nCRT
134 tumor biopsy not available
11 no tumor in biopsy sample
160 biopsy samples
6 tumor recurrent
10 no TRG data
3 no surgery
141 analysed
